# Case for diagnosis. Phagedenic ulcer on the thorax^⋆^^⋆⋆^

**DOI:** 10.1016/j.abd.2020.03.012

**Published:** 2020-08-16

**Authors:** Isabella Lemos Baltazar, Flávia Regina Ferreira, Mariana Galhardo Tressino, Fernanda da Rocha Gonçalves

**Affiliations:** a Dermatology Service, Hospital Municipal Universitário de Taubaté, Taubaté, SP, Brazil; b Department of Medicine, Universidade de Taubaté, Taubaté, SP, Brazil; c Hospital do Servidor Público Estadual, São Paulo, SP, Brazil

**Keywords:** Breast, Neoplasms, Paget's disease, mammary

## Abstract

Paget's disease is a rare disorder of the nipple and/or the areola that is characterized by an erythematosquamous lesion and is often associated with *in situ* or invasive breast carcinoma. The authors present an atypical, exuberant case that had evolved over eight years, emphasizing the importance of early diagnosis.

## Case report

**Figure 1:**
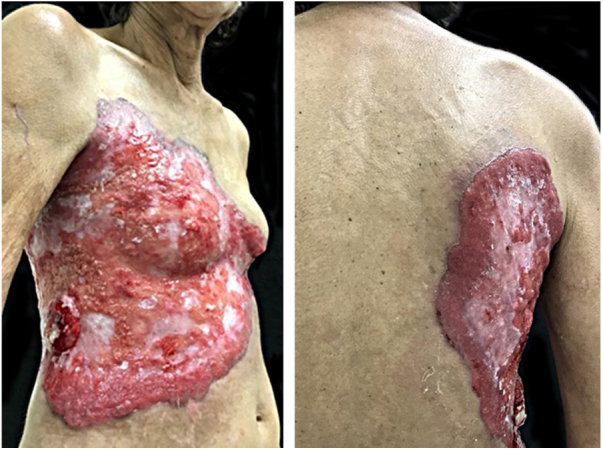
Chest and abdomen on the right side: extensive ulcer with infiltrated, irregular, erythematous-violaceous borders. Lateral of the trunk: vegetative erythemato-ulcerated tumor.

**Figure 2:**
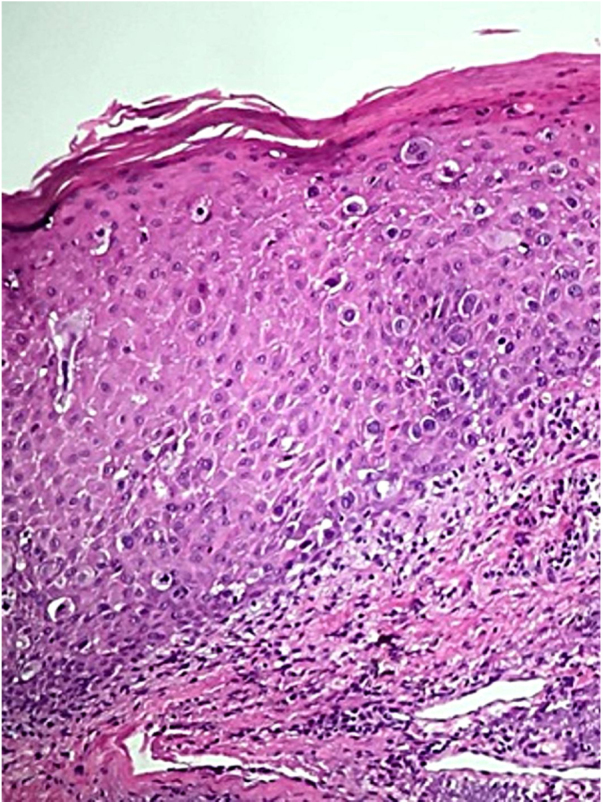
Epidermis showing infiltration by atypical epithelial cells with broad and clear cytoplasm (Hematoxylin & eosin, x100).

**Figure 3:**
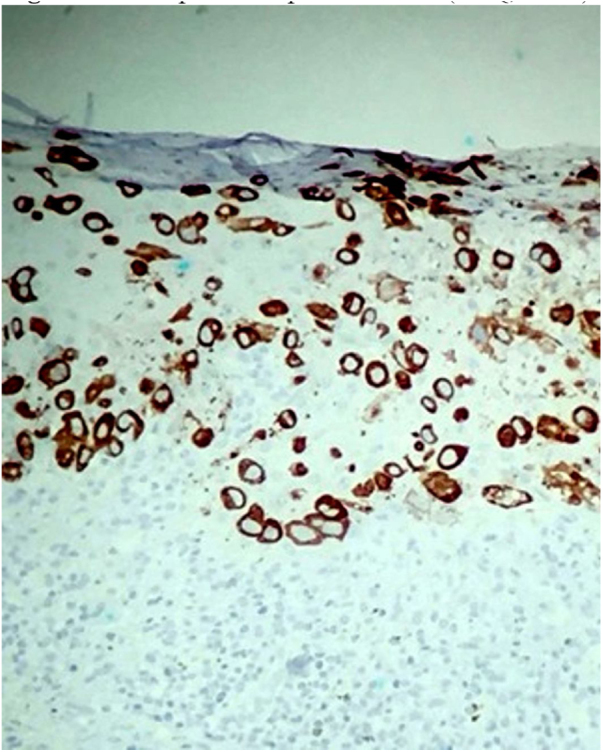
CK7-positive epithelial cells (Immunohistochemistry, x100).

A female patient, 64 years old, mixed race, complained of a lesion in the right nipple for eight years. On examination, she presented a phagedenic ulcer, of approximately 45 cm in the largest diameter, with infiltrated and irregular erythematous-violaceous borders, extending from the left breast, across the right hemithorax, where tumoral lesion and extension of the ulcer to the right dorsum were observed (Fig. 1). She denied personal and family history of breast cancer. Incisional biopsies at the border of the ulceration and in the tumor showed evidence of malignant neoplasia restricted to the epidermis, exulcerated, and characterized by the presence of numerous individualized atypical epidermal cells, with large nuclei and clear cytoplasm (Fig. 2). The immunohistochemical panel was negative for estrogen and progesterone receptors and revealed a 3+/3 positive (score 3+) membrane pattern for c-erbB-2 and CK7 (Fig. 3).

## What is your diagnosis?

a) Squamous cell carcinoma
b) Skin metastasis from breast cancer
c) Paget's disease
d) Pyoderma gangrenosum

## Discussion

Paget's disease (PD) of the breast is an uncommon disease, accounting for 0.5–5% of all breast carcinomas.^1^ It is usually observed in postmenopausal women.^2^

Clinically, it presents as an erythematous or eczematoid lesion, with irregular borders, usually limited to the nipple or spreading to the areola; in advanced cases, it can also involve the surrounding skin. Pain or itching are frequent.^3^

It is associated with underlying breast cancer in 92–100% of the cases.^4^, ^5^ Approximately 50% present a palpable mass in the breast, usually an associated carcinoma.^3^, ^6^ Patients who do not have a palpable mass are likely to have ductal carcinoma *in situ.*^7^

Biopsy for histopathological and immunohistochemical examination is the gold standard for diagnosing this condition.^8^

Histologically, the classic pattern comprises large oval or round intraepidermal cells with clear and broad cytoplasm, and pleomorphic and hyperchromatic nuclei. Paget cells are most often located in the lower layers of the epidermis, as single cells or as clusters of cells that form structures similar to glands or nests.^1^, ^2^

The immunohistochemical profile corroborates the diagnosis and assists in the differentiation from other entities; furthermore, it defines the cell of origin in PD. Paget cells are positive for CK7 in almost all cases, and are not reactive with CK20. They are often negative for estrogen and progesterone receptors, because the underlying carcinomas tend to be poorly differentiated.^1^, ^8^, ^9^ The c-erbB-2 oncoprotein is overexpressed in the majority (>90%) of cases of mammary PD, a finding also observed in the present case. In many patients, a correlation is observed between the positivity of c-erbB-2 oncoprotein in the Paget cells and in the underlying intraductal *in situ* or invasive breast carcinoma.^10^

Due to the clinical similarity of PD with other dermatoses, the differential diagnosis must include atopic or contact dermatitis, Bowen's disease, superficial basal cell carcinoma or superficial spreading melanoma, and psoriasis, diagnostic hypotheses that were not applicable to the present case.^6^

As a general rule, in any chronic dermatosis of the nipple or areola, the skin should be examined histologically for a conclusive diagnosis.^6^ The dermatologist and the gynecologist play a fundamental role in the early diagnosis of PD; by paying due attention to the patient complaints and performing a complete physical examination, they can impact the prognosis of these patients.

The present case was referred for specialized oncologic follow-up.

## Financial support

None declared.

## Authors’ contributions

Isabella Lemos Baltazar: Approval of the final version of the manuscript; elaboration and writing of the manuscript; intellectual participation in propaedeutic and/or therapeutic conduct of studied cases; critical review of the literature; critical review of the manuscript.

Flávia Regina Ferreira: Approval of the final version of the manuscript; elaboration and writing of the manuscript; intellectual participation in propaedeutic and/or therapeutic conduct of studied cases; critical review of the literature; critical review of the manuscript.

Mariana Galhardo Tressino: Approval of the final version of the manuscript; elaboration and writing of the manuscript.

Fernanda da Rocha Gonçalves: Approval of the final version of the manuscript; intellectual participation in propaedeutic and/or therapeutic conduct of studied cases; critical review of the manuscript.

## Conflicts of interest

None declared.
